# Editorial: Muscle plasticity: Regulation of adaptive changes in muscles and mitochondrial dynamics

**DOI:** 10.3389/fphys.2023.1192759

**Published:** 2023-04-11

**Authors:** Prasanna Katti, Yuho Kim, N. Ravi Sundaresan, Gilles Gouspillou, Antentor Hinton

**Affiliations:** ^1^ National Heart, Lung and Blood Institute, National Institutes of Health, Bethesda, MD, United States; ^2^ Department of Physical Therapy and Kinesiology, University of Massachusetts Lowell, Lowell, MA, United States; ^3^ Cardiovascular and Muscle Research Laboratory, Department of Microbiology and Cell Biology, Indian Institute of Science, Bengaluru, India; ^4^ Department of Exercise Science, Université du Québec à Montréal, Montréal, QC, Canada; ^5^ Department of Molecular Physiology and Biophysics, Vanderbilt University, Nashville, TN, United States

**Keywords:** mitochondria, sarcopenia, TCAP-1, HIIT, resistant exercise, muscles

## Introduction

Skeletal muscle comprises long, cylindrical muscle fibers that are multinucleated and striated, i.e., they appear striped under a microscope (Frontera and Ochala, 2015). Skeletal muscles are under voluntary control and mediate walking, running, jumping, breathing, and lifting weights. They work with tendons, which attach muscle to bone, and neurons, which signal the muscle fibers to contract (Widmaier and Vander, 2019). Skeletal muscles also play key roles in glucose, protein, and lipid metabolism, and they contribute approximately 40% of total body weight and 50%–75% of all proteins in the body (Frontera and Ochala, 2015; Saladin et al., 2018). Muscle mass depends on the balance between protein synthesis and degradation. Both processes are sensitive to nutritional status, hormonal equilibrium, physical activity/exercise, and injury or disease (Frontera and Ochala, 2015; Saladin et al., 2018).

Skeletal muscles can modify their size in response to use (Frontera and Ochala, 2015; Jorgenson et al., 2020) and this impressive plasticity in metabolism and function can be linked to organelles, such as mitochondria, peroxisomes, and endoplasmic reticulum. While researchers have made significant strides in understanding the regulation of muscle mass and mitochondrial metabolism in physiological and pathophysiological conditions, further investigation is needed to uncover the mechanisms underlying muscle plasticity. This editorial article introduces our Research Topic, which aims to provide new insights into muscle plasticity and mitochondrial metabolism. The featured articles showcase the role of teneurin C-terminal associated peptide (TCAP-1) in regulating mitochondrial metabolism and muscle contraction (Hogg et al.), introduce a novel method to rapidly isolate functional mitochondria (Younis et al.), and review the role of exercise and mitochondrial mechanisms in sarcopenia (Alizadeh Pahlavani et al.) and the effect of acute exercise on muscle protein turnover (Hajj-Boutros et al.).

## Highlights

Hogg et al. unveiled a new mechanism for regulating skeletal muscle dynamics through TCAP-1 action. TCAP-1 modulates energy metabolism in skeletal muscle via an insulin-independent pathway, which affects contractile kinetics through changes in Ca^2+^ dynamics and ATP production. The authors identified interactions between TCAP-1 and LPHN-1 and LPHN-3, which activates G-protein-coupled PLC, leading to subsequent changes in intracellular IP3 and DAG levels. Additionally, TCAP-1 increases cellular energy availability by promoting glucose importation into cells, likely due to increased GLUT4 expression.

Younis et al. presented a new method for rapidly and efficiently isolating intact, respiring mitochondria from cultured skeletal muscle cells and murine tissue. This protocol offers high yield and purity of mitochondria with minimal contamination from other cell fractions by using nitrogen cavitation and rapid differential centrifugation to lyse cell membranes and isolate mitochondria. Further, this method allows for low variation in respiratory control ratio, with a ± 0.2 and 0.8 standard error of mean in isolated mitochondria from tissue and cells, respectively, making it highly suitable for high-resolution respiratory studies. This new protocol offers a significant advantage for researchers working with skeletal muscle tissues, particularly for respiratory studies and proteome analysis. The protocol will facilitate downstream mitochondrial evaluation, with important implications for advancing our understanding of human health and disease.

Sarcopenia, the loss of muscle mass and function in the elderly, significantly contributes to reduced quality of life in older individuals (Romanello, 2020). Alizadeh Pahlavani et al. provide a thorough explanation of exercise intervention and mitochondrial mechanisms in sarcopenia muscles. This article reviews the evidence linking physical inactivity, reduced antioxidant defenses and impaired mitochondrial function in sarcopenia. This review also explains that age-associated disruption in multiple mitochondrial quality control processes, including mitochondrial biogenesis, fusion, fission, and mitophagy are important contributors to sarcopenia. This review highlights exercise as the most effective way to improve mitochondrial function and biogenesis, as well as to reduce oxidative stress in aging muscles. Exercise can activate signals that increase the expression of critical genes involved in mitochondrial biogenesis (i.e., PGC-1α, PGC-1β, TFAM, and Nrf-1), thereby improving mitochondrial biogenesis, which is essential for preserving muscle function in the elderly. Alizadeh Pahlavani et al. also review the evidence that exercise positively impacts mitochondrial dynamics (fusion and fission) and mitophagy, which are mechanisms that likely contribute to improving mitochondrial and muscle function in the elderly. Overall, the article describes in detail how sarcopenia is a complex condition involving oxidative stress, impaired mitochondrial function, and impaired mitochondrial quality control processes, and illustrates how exercise can combat sarcopenia, by increasing both mitochondrial and muscle quality, thus improving overall health and wellbeing.

The review article by Hajj-Boutros et al. details the mechanisms involved in muscle protein turnover after acute exercise, focusing on the acute effects of resistance exercise (RE) and high-intensity interval training (HIIT) on muscle protein synthesis and breakdown. The review notes that older adults generally have a reduced response to exercise compared with younger individuals, with a subtle increase in their muscle protein synthesis (MPS) rate following an acute bout of exercise. The review describes the effect of RE on MPS and the signaling pathways involved, such as insulin-like growth factor-1 (IGF-1) and its downstream targets, which increase MPS. RE is also a potential activator of the integrin pathway and is involved in MPS through extracellular signal-regulated kinase (ERK1/2). Reduced stimulation of both pathways might underlie diminished or delayed MPS in elderly adults. Elderly adults can still gain muscle mass through RE, but the process is slower due to a decreased ability to respond to exercise stimuli. RE and endurance exercise (EE) result in different muscular and cellular adaptations and the duration of exercise determines the positive effects induced by RE and EE. In older adults, HIIT can induce muscle hypertrophy to a certain degree by potentially stimulating MPS, while RE + HIIT can improve muscle strength, functional capacity, and aerobic capacity. Further, the combination of RE + HIIT significantly increased markers of mitochondrial biogenesis compared to RE alone, but downstream signaling molecules were not affected, indicating attenuated protein synthesis. Overall, examining the individual response to exercise has become more complex, especially in aged populations, and predicting how people will respond to exercise is challenging. This review suggests further research is needed to examine the effects of RE + HIIT on changes in muscle mass.

## Closing comments

This Research Topic provides fundamental insights into the determinants of muscle plasticity and mitochondrial activity. These research findings hold significant promise for developing therapeutic strategies to promote and enhance muscle health. Furthermore, this Research Topic contributes to understanding the intricate molecular mechanisms that govern healthy mitochondrial function in muscle and the complex processes underlying sarcopenia and protein turnover in skeletal muscle.
